# Improved Leptin Sensitivity as a Potential Candidate Responsible for the Spontaneous Food Restriction of the Lou/C Rat

**DOI:** 10.1371/journal.pone.0073452

**Published:** 2013-09-06

**Authors:** Christelle Veyrat-Durebex, Anne-Laure Poher, Aurélie Caillon, Emmanuel Somm, Philippe Vallet, Yves Charnay, Françoise Rohner-Jeanrenaud

**Affiliations:** 1 Laboratory of Metabolism, Department of Internal Medicine Specialties, Faculty of Medicine, University of Geneva, Geneva, Switzerland; 2 Division of Development and Growth, Department of Pediatrics, Geneva University Hospital, Geneva, Switzerland; 3 Department of Psychiatry, Geneva University Hospital, Geneva, Switzerland; Wayne State University, United States of America

## Abstract

The Lou/C rat, an inbred strain of Wistar origin, was described as a model of resistance to age- and diet-induced obesity. Although such a resistance involves many metabolic parameters described in our previous studies, Lou/C rats also exhibit a spontaneous food restriction due to decreased food consumption during the nocturnal period. We then attempted to delineate the leptin sensitivity and mechanisms implicated in this strain, using different protocols of acute central and peripheral leptin administration. A first analysis of the meal patterns revealed that Lou/C rats eat smaller meals, without any change in meal number compared to age-matched Wistar animals. Although the expression of the recognized leptin transporters (leptin receptors and megalin) measured in the choroid plexus was normal in Lou/C rats, the decreased triglyceridemia observed in these animals is compatible with an increased leptin transport across the blood brain barrier. Improved hypothalamic leptin signaling in Lou/C rats was also suggested by the higher pSTAT3/STAT3 (signal transducer and activator of transcription 3) ratio observed following acute peripheral leptin administration, as well as by the lower hypothalamic mRNA expression of the suppressor of cytokine signaling 3 (SOCS3), known to downregulate leptin signaling. To conclude, spontaneous hypophagia of Lou/C rats appears to be related to improved leptin sensitivity. The main mechanism underlying such a phenomenon consists in improved leptin signaling through the Ob-Rb leptin receptor isoform, which seems to consequently lead to overexpression of brain-derived neurotrophic factor (BDNF) and thyrotropin-releasing hormone (TRH).

## Introduction

Since many years, numerous studies tried to identify neuroendocrine mechanisms involved in the regulation of food intake and body weight gain in normal, as well as in pathological conditions, such as in rodents presenting different susceptibilities to develop obesity [1]. Among them, the Lou/C rat, an inbred strain of Wistar origin, was described as being resistant to obesity development with age [2–4] or in response to a high fat diet [5,6]. Interestingly, this rodent model also exhibits a reduced daily food intake under standard [4], self-selection [7] and a high fat diet [5] compared to age-matched Wistar rats. Such changes are accompanied by a lower body fat mass [4,5] and low circulating leptin levels [4,5,8] in Lou/C rats, similarly to what was observed during long-term moderate (40%) caloric restriction [9]. Of note, low plasma leptin levels are also observed in dietary-resistant (DR) Sprague Dawley rats compared to diet-induced obese (DIO) animals exhibiting high leptin levels [10]. In DIO rats, reduced central leptin sensitivity seems to partially explain their propensity to develop obesity under a high energy diet, since both NPY mRNA expression and food intake were poorly modulated after 4 weeks of food restriction and intracerebroventricular leptin injection, respectively [11].

Following a first experiment bearing on the analysis of the meal pattern in Wistar and Lou/C rats, the aim of the present study was to compare leptin sensitivity between the two groups. For this purpose, different protocols of acute leptin treatment (centrally, peripherally, in addition to Glucagon-like peptide 1, GLP-1) were used to measure the leptin effects on food intake, as well as different parameters involved in leptin action. Considering the literature, two hypotheses have received considerable attention concerning the occurrence of leptin resistance during the development of obesity: 1) failure of circulating leptin to reach its central targets [12] and 2) impaired intracellular leptin signaling [13]. Leptin transport through the blood-brain-barrier (BBB) was shown to be blunted by high levels of circulating triglycerides [14,15] or by leptin transporter (Ob-R) saturation [16,17]. With regard to leptin signaling occurring through the phosphorylation of the signal transducer and activator of transcription (STAT3), it may be blunted by endoplasmic reticulum (ER) stress [18], central inflammation and/or gliosis [19]. Different parameters involved in these pathways were thus evaluated in adult Lou/C rats. Finally, the endpoints of leptin signaling were studied by measuring the mRNA expression of several direct targets of central leptin, such as the suppressor of cytokine signaling 3 (SOCS3), known to downregulate leptin signaling, the orexigenic neuropeptide Y (NPY) [20], anorexigenic factors such as proopiomelanocortin (POMC) [20] and brain-derived neurotrophic factor (BDNF) [21], or metabolic sensors such as thyrotropin-releasing hormone (TRH) [22].

## Materials and Methods

### Ethics Statement

All procedures were performed in accordance with and approved by the Institutional Ethical Committee of Animal Care in Geneva and Cantonal Veterinary Office (experiment ID 1034/3064/2-R).

### Animals

3 month-old male Lou/C and Wistar rats were purchased from Harlan UK Limited (Oxon, UK) and Charles River (L’Arbresle, France), respectively. They were housed under controlled temperature (22°C) and lighting (light on: 07.00-19.00 h) with free access to water and to standard laboratory diet (2.61 kcal/g; RM1, SDS, Essex, UK). At the end of the experiments, rats were euthanatized using isoflurane anesthesia (Halocarbon Laboratories, River Edge, NJ) and rapid decapitation. In some cases, this was carried out 30 min after i.p. injection of leptin i.p. (2 mg/kg) (leptin challenge) or vehicle (NaCl 0.9%) (basal state). Blood was sampled and tissues were rapidly removed, freeze-clamped and stored at -80°C.

### Analyses of meal patterns

Microstructure of meals was determined using the 12-cage LabMaster system (TSE Systems GmbH, Berlin, Germany) of the Small Animal Phenotyping Core Facility (CMU, University of Geneva, Geneva) under controlled temperature (22 + 1°C) and lighting (light on: 07.00-19.00 h). Food and drinking behaviors were studied using highly sensitive feeding and drinking sensors for automated online measurements. Before recording (2 x 24hrs), animals were allowed a 7-day acclimatization period in training cages. Food intake analyses were realized with a fixed sequence analysis (intermeal interval) of 15 minutes, i.e. a meal is ended when no further removal has occurred before the end of the intermeal interval. Here, all meals that occurred during the whole trial are listed chronologically, allowing for the determination of meals occurring simultaneously in both Wistar and Lou/C rats (common meals).

### Body composition

An EchoMRI-700™ quantitative nuclear magnetic resonance analyzer (Echo Medical Systems, Houston, TX) was used to measure body composition (total fat and lean body mass).

### Intracerebroventricular (i.c.v.) leptin injection

Rats were individually housed and submitted to surgical procedures. They were anesthetized with intramuscular injection of ketamine-xylasine (Ketalar®-Rompun®, Parke-Davis and Bayer, Leverkusen, Switzerland) at 40 and 9 mg/kg, respectively and implanted with a cannula in the right lateral cerebral ventricle, as already described [23]. After one week of recovery, the drinking response to i.c.v. injection of Angiotensin II (5 ng/μl) (Novabiochem, Läufelfingen, Switzerland) was measured to confirm the correct placement of the cannula. In order to test the central response to leptin, Lou/C and Wistar rats were weighed, deprived from food 4hrs before the onset of the dark cycle (15: 00 hrs) and injected i.c.v. with human leptin (10 μg/rat, 4 μl) (H-3424, Bachem, Bubendorf, Switzerland) or its vehicle (NaCl 0.9%, 4 μl) [24]. At 19: 00 hrs, preweighed amounts of food were returned into the cages and food intake was measured 0.5, 1, 2, 4 and 12hrs later. The following week, rats were once again treated with i.c.v. injections of leptin (10 μg/rat) or vehicle at 15: 00 hrs and killed 30 minutes later by rapid decapitation under isoflurane anesthesia.

### Peripheral (i.p.) leptin injection

Intraperitoneal (i.p.) injections of human leptin (0.5, 1 and 2 mg/kg) (Bachem, as above) or vehicle (NaCl 0.9%) were administered 1h before the start of the dark cycle (18: 00 hrs), time at which food was removed. At 19: 00 hrs, preweighed amounts of food were returned into the cages and food intake was measured 0.5, 1, 2, 4 and 12 hrs later.

### Effect of peripheral leptin on the anorexigenic response to GLP-1

Rats were randomly assigned to saline/saline, saline/GLP-1, leptin/saline, or leptin/GLP-1 groups. As previously described [25], food was removed 4 hrs before the onset of the dark cycle. A first i.p. injection of either vehicle (NaCl 0.9%) or leptin (0.5 mg/kg, subthreshold dose that does not reduce food intake) was administered 1 h before the start of the dark cycle (18: 00 hrs). At 19: 00 hrs, rats received a second injection of either vehicle (NaCl 0.9%) or GLP-1 (100 µg/kg). Preweighed amounts of food were then returned into the cages and food intake was measured 0.5, 1, 2, 4 and 12 hrs later.

### Plasma measurements

Plasma leptin levels were determined using a double antibody radioimmunoassay (RIA) with a 3% intra-assay coefficient of variation (Linco Research Inc., St Charles, MO). Plasma insulin levels were measured using a RIA, as previously described [26]. An enzymatic commercial kit, PAP 150 (BioMerieux SA, Marcy l’Etoile, France) was used to determine plasma triglyceride levels.

### Tissue processing and Reverse Transcription-Polymerase Chain Reaction (RT-PCR)

Total RNA from hypothalamus was extracted with the RNeasy Mini Kit® according to the manufacturer’s protocol (Qiagen, Basel, Switzerland). Two micrograms of total RNA were reverse-transcribed using 400 units of Moloney Murine Leukemia Virus Reverse Transcriptase (Invitrogen, Basel, Switzerland), in the presence of 1 U/μl RNAsin (Promega Corp, Madison, WI), 0.2 μg of random primers oligo(dN) 6 (Promega), 2 mM dNTP and 20 µM of DTT (Invitrogen). The expression of the cDNAs was determined by quantitative real-time PCR using an ABI StepOne Plus Sequence Detection System (Applera Europe, Rotkreuz, Switzerland). PCR products were quantified using the Master SYBR Green mix (Applera Europe). Results were normalized to the expression levels of the 36b4 housekeeping gene. Primers were designed using the Primer Express software (Applera Europe), chosen when possible on both sides of an intron to avoid potential amplification of contaminating genomic DNA. Oligos were used at 200 to 300 nM (Table 1).

**Table 1:** Primer sequences used for qPCR.

Gene	Ascension Number	Forward primer sequence	Reverse primer sequence
AgRP	NM_033650.1	5’-ACAGAACCGCGAGTCTCGTT	5’-ACAGGTCGCAGCAAGGTACCT
BDNF	NM_012513.3	5’-CCATAAGGACGCGGACTTGT	5’-AGCAGAGGAGGCTCCAAAGG
CART	NM_017110.1	5’-GGATGATGCGTCCCATGAG	5’-CAGCGCTTCAATCTGCAACA
GFAP	NM_017009.2	5’-CCATGAGGAGGAAGTTCGAGAA	5’-GGTCTGGCTTGGCCACAT
IL1β	NM_031512.2	5’-GGCAACTGTCCCTGAACTCAA	5’-GCCTCAAAGAACAGGTCATTCTC
IL6	NM_012589.1	5’-ATATGTTCTCAGGGAGATCTTGGAA	5’-TGCATCATCGCTGTTCATACAA
MC4-R	NM_013099.2	5’-CGCGCTCCAGTACCATAACA	5’-CAAGCTGCCCAGATACAACTGA
NPY	NM_012614.1	5’-TGATGCTAGGTAACAAACGAATGG	5’-GCCAGAATGCCCAAACACA
Ob-Rb	D_85558	5’-AACCTGTGAGGATGAGTGTCAGAGT	5’- CCTTGCTCTTCATCAGTTTCCA
Orexin	AF_041241.1	5’-AGATACCATCTCTCCGGATTGC	5’-CCAGGGAACCTTTGTAGAAGGA
POMC	NM_139326.2	5’-TGCCTTTCCGCGACAGA	5’-CCTGAGCGACTGTAGCAGAATCT
PTP-1B	NM_013016	5’-CGAGGGTGCAAAGTTCATCA	5’-CCAGGTCTTCATGGGAAAGC
SOCS3	NM_053565.1	5’-CCTCAAGACCTTCAGCTCCAA	5’-TCCGCTCTCCTGCAGCTT
STAT3	NM_012747.2	5’-CTGGCGCCTTGGATTGAG	5’-CGATCTCGCCCAAGAGGTT
TNFα	NM_012675.3	5’-GACCCTCACACTCAGATCATCTTCT	5’-TCCGCTTGGTGGTTTGCTA
TRH	NM_013046.2	5’-GCTGCCTTAGACTCCTGGATCA	5’-TCTTCAGCTTCAATGTCTTTTTCCT
36b4	AC_130745.2	5’-TTCCCACTGGCTGAAAAGGT	5’-CGCAGCCGCAAATGC

### Western blot

Frozen tissues were mechanically homogenized in ice-cold CHAPS 1% for hypothalami or RIPA buffer for choroid plexus, containing protease inhibitors (Complete Mini, Roche Diagnostics). Proteins were separated by SDS-10% polyacrylamide gel electrophoresis. After transfer onto nitrocellulose membranes and blocking period (milk 5% TBS tween 0.1%, 1h), blots were incubated with purified anti-STAT3 (1:2,000), anti-pSTAT3 (1:2,000), anti-FoxO1 (1:1,000) (Cell Signaling Tech. Inc., Danvers, MA), anti-megalin (1:3,000) (Santa Cruz, Biotechnology, Inc., Santa Cruz, CA), anti-JAK2 (1:500), anti-pJAK2 (1:500) (Millipore, Zug, Switzerland), and anti-actin (1:100,000) (Chemicon International Inc., Billerica, MA) antibodies overnight at 4°C. Detection was performed using horseradish peroxidase-conjugated secondary antibodies (1:3,000) and an enhanced chemiluminescence (ECL) detection system (GE Healthcare, Europe GmbH, Glattbrugg, Switzerland). The results were then quantified using the ChemiDoc^TM^ XRS from BIO-RAD Laboratories (Reinach, Switzerland) and the Quantity One^TM^ software.

### Data analyses

Results are expressed as mean ± SEM. Comparisons between Lou/C and Wistar rats for feeding patterns and metabolic parameters were performed using the Student’s *t* test (SPSS Inc., Chicago, IL). The effect of leptin/GLP-1 injections on food intake in both strains was analyzed using the parametric one-way analysis of variance, followed by the Bartlett’s *a posteriori* test. Statistical significance was established at p<0.05.

## Results

### Analysis of meal patterns suggests higher leptin sensitivity in Lou/C rats

**Figure 1:**
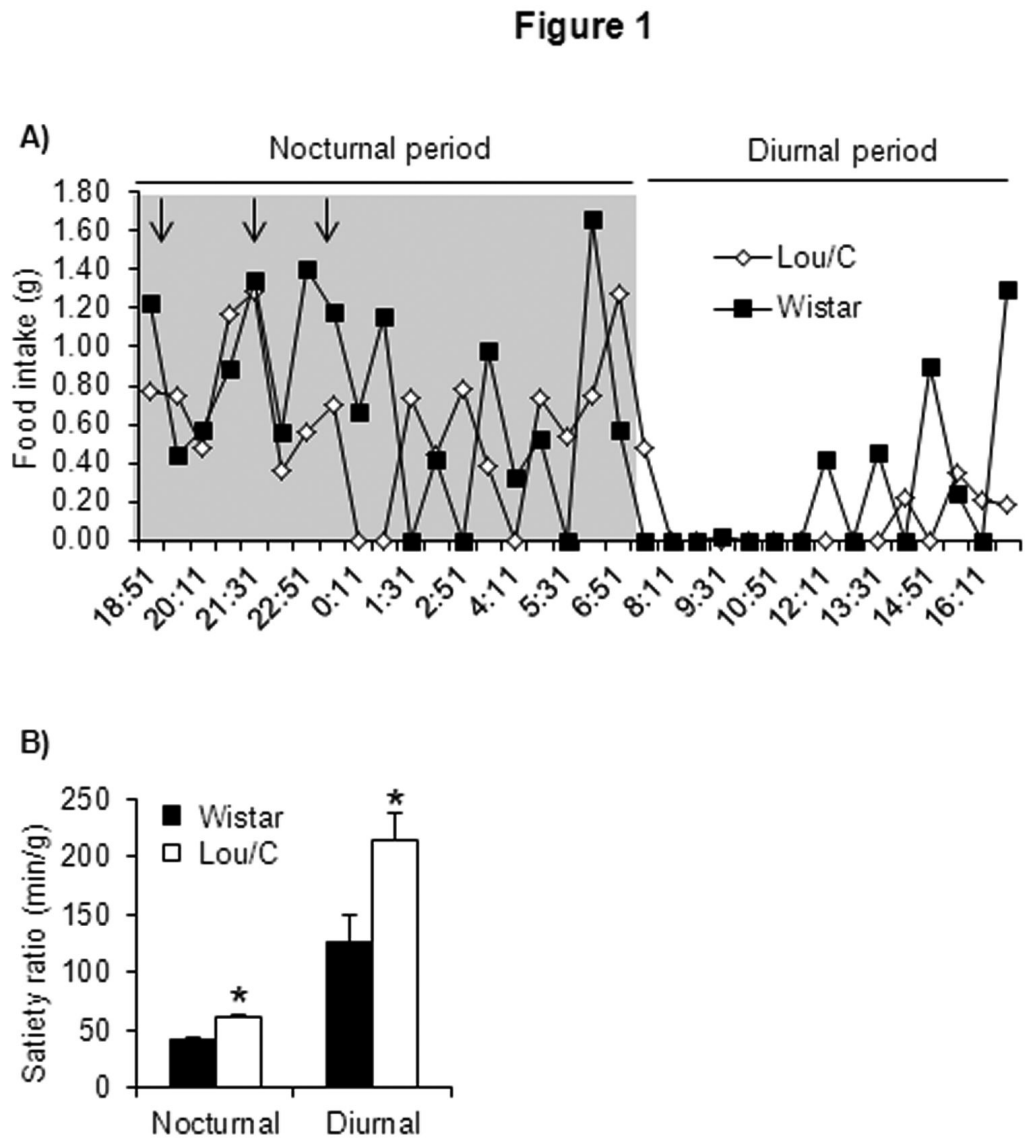
Feeding behavior and microstructure of meals of Lou/C and Wistar rats. A) Profile of meal patterns over one day of recording (nocturnal (19:00-7:00) and diurnal (7:00-19:00) periods). Black arrows: common meals in Lou/C and Wistar rats. B) Satiety ratio (Intermeal interval/quantity of food eaten) in minutes/grams, during nocturnal and diurnal periods. Values are mean + SEM of 6 animals per group. *p<0.05 compared to Wistar rats using the Student’s *t* test.

As previously described [4], 3 month-old Lou/C rats have a lighter body weight than age-matched Wistar rats, with a higher percentage of lean mass and a lower percentage of fat mass (Table 2). The latter is in keeping with the observed lower plasma leptin levels in the Lou/C than in the Wistar group (Table 2). No significant intergroup difference was noted for insulinemia, while plasma triglyceride levels were decreased in Lou/C compared to Wistar animals. These metabolic changes were accompanied by subtle modifications in the detailed pattern of food intake. Thus, analysis of the microstructure of meals using the LabMaster system showed the presence of well-defined common meals in Lou/C and Wistar rats, at extinction of the light (19: 07 hrs) and around 2 (20: 47 hrs) and 4hrs (23: 07 hrs) later (Figure 1A, arrows). A common meal was also observed just before the lights off. However, Lou/C rats exhibited a lower daily food intake than Wistar animals due to decreased food consumption during the nocturnal period (Table 3). This was specifically related to reduced meal size (g), without any change in meal number (Table 3). Interestingly, a higher satiety ratio (intermeal interval/meal size; min/g) during both the day and the night was observed in Lou/C rats (Figure 1B). As the main alterations in meal pattern in Lou/C rats is similar to the changes elicited by central and peripheral leptin administration (for review, 27), we hypothesized that leptin sensitivity is enhanced in this group of animals compared to Wistar rats.

**Table 2:** Body composition and metabolic parameters in 3 month-old Lou/C and Wistar rats.

	Wistar	Lou/C	Student’s t test
Body weight (g)	343.2 + 5.1	228.3 + 3.9*	p=0.0001
% fat mass	12.7 + 0.8	10.3 + 0.4*	p=0.0294
% lean mass	74.9 + 0.8	78.9 + 0.4*	p=0.0014
% total water	60.3 + 0.7	62.9 + 0.4*	p=0.0064
Leptin (ng/mL) 15:00 hrs	6.66 + 1.24	1.44 + 0.24*	p=0.0011
Leptin (ng/mL) 18:00 hrs	3.78 + 0.50	1.75 + 0.22*	p=0.0004
Insulin (ng/mL) 15:00 hrs	2.36 + 0.45	1.45 + 0.25	NS
Insulin (ng/mL) 18:00 hrs	3.03 + 0.37	2.22 + 0.30	NS
Triglycerides (mg/dL) 15:00 hrs	47.3 + 4.7	26.2 + 1.2*	p=0.0004

Values are mean + SEM.

**Table 3:** Microstructure of meals in 3 month-old Lou/C and Wistar rats.

		Wistar	Lou/C	Student’s t test
**Food Intake (g)**	Nocturnal	16.40 + 0.90	12.05 + 0.63	p=0.052
	Diurnal	2.39 + 0.44	2.16 + 0.17	NS
	24hrs	18.79 + 0.88	14.21 + 0.61*	p=0.032
**Meal size (g)**	Nocturnal	1.73 + 0.11	1.32 + 0.04*	p=0.005
	Diurnal	0.95 + 0.13	0.90 + 0.07	NS
**Meal Number**	Nocturnal	9.5 + 0.7	9.3 + 0.6	NS
	Diurnal	2.5 + 0.2	2.3 + 0.2	NS
	24hrs	14.0 + 0.7	14.2 + 0.4	NS

Values are mean + SEM.

### Central and peripheral injections of leptin confirm higher leptin sensitivity in Lou/C rats

**Figure 2:**
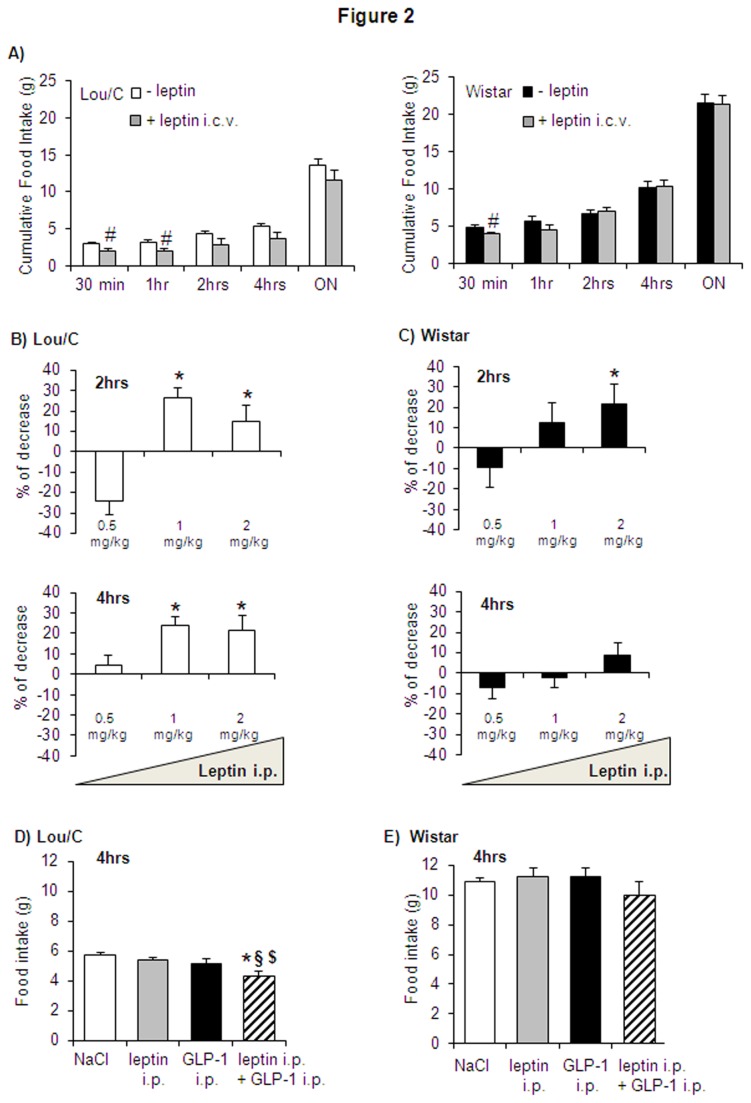
Effect of central (i.c.v.) and peripheral (i.p.) injection of leptin on food intake and on anorectic effect of GLP-1 in Lou/C and Wistar rats. A) Effect of i.c.v. leptin injection (10 µg, 15: 00) on food intake 30 minutes, 1, 2, 4 hours after lights off, and on overnight (ON) food intake in Lou/C and Wistar rats. Values are mean + SEM of 8 rats per group. B and C) Percent decrease in food intake in Lou/C and Wistar rats, 2 and 4 hours after i.p. injection of leptin (0.5, 1 and 2 mg/kg, 19: 00). D and E) Effect of i.p. co-injection of leptin (0.5 mg/kg, 18: 00) and GLP-1 (100 µg/kg, 19: 00) on total food intake, 4 hours after GLP-1 injection. Values are mean + SEM of 6-8 animals per group. *p<0.05 compared to vehicle- (NaCl 0.9%) treated rats; ^§^ p<0.05 compared to leptin-treated rats; ^$^ p<0.05 compared to GLP-1-treated rats using Student’*t* test.

To support the hypothesis of higher leptin sensitivity in Lou/C than in Wistar rats, different protocols of central (i.c.v.) or peripheral (i.p.) leptin injection were tested. First, food-deprived animals received an i.c.v. injection of leptin (10 µg) 4 hours before the dark period (15: 00 hrs). In Lou/C rats, a 34% and 38% inhibitory effect of leptin on food intake was observed 30 minutes and 1hr after the lights off, respectively (Figure 2A). This effect was less marked in Wistar rats (18% of inhibition), being only observed after 30 min.

In a second experiment, leptin was administered i.p. at different doses (0.5, 1 and 2 mg/kg) in Lou/C and Wistar rats, 1 h before the onset of the dark cycle. In the Lou/C group, the maximal anorexigenic effect of leptin on food intake (26.5 + 5.1%) was observed 2 hrs after extinction of the light, at a dose of 1 mg/kg (Figure 2B). Wistar rats appeared to be less sensitive to leptin, exhibiting a 21.6 + 7.7% decrease in food intake 2hrs after extinction of the light, in response to 2 mg/kg leptin (Figure 2C).

Finally, the potency of leptin to improve the satiety effect of GLP-1, a product of the preproglucagon gene initiating satiety in the nucleus of the solitary tract (NTS) of the hindbrain [28,29], was tested. To this end, leptin (0.5 mg/kg) was injected 1 h before GLP-1 (100 µg/kg i.p., lights off). Neither leptin, nor GLP-1 used at the doses chosen, had an effect on food intake when injected alone (Figure 2D and 2E). No differences in meal patterns were observed 30 minutes and 2 hrs after the lights off (data not shown). However, as shown on Figure 2D, leptin was able to potentiate the effect of GLP-1 in Lou/C rats with a maximal effect seen 4 hrs after extinction of the lights (F_2,23_ = 4.931, p = 0.0176) (Figure 2D). This was not observed in the Wistar group (Figure 2E).

### Potential mechanisms underlying the enhanced leptin sensitivity of Lou/C rats

The next aim of this study was to attempt delineating the mechanisms involved in enhanced leptin sensitivity of Lou/C rats. This was done by evaluating some parameters of the following processes known to modulate leptin sensitivity: Leptin transport through the blood brain barrier (BBB); Activation of the neuronal leptin receptor; Expression levels of various factors known to influence the neuronal leptin receptor activation; Gene expression levels of the main hypothalamic neuropeptides involved in the anorexigenic effect of leptin. Some measurements pertaining to the first two sequential steps of leptin action needed to be evaluated in the presence of leptin, injected i.p. at a dose of 2 mg/kg, 30 min before sacrifice (leptin transport through megalin, activation of STAT3), whereas those involved in the last steps of leptin action were evaluated under basal conditions (i.e. without leptin challenge) to get a general overview of the leptin circuit activity.

**Figure 3:**
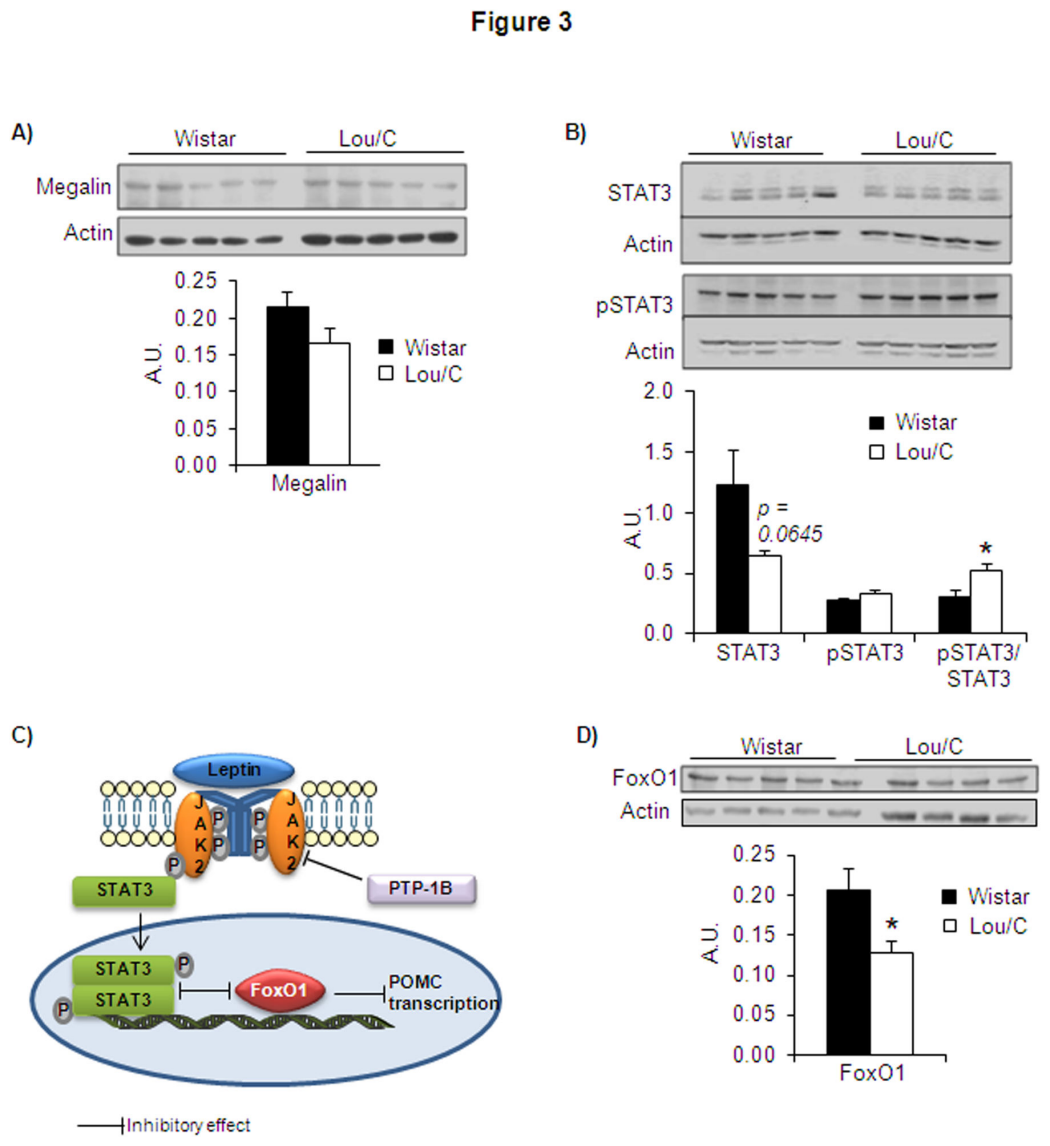
Levels of megalin and STAT3 and pSTAT3 protein expression in Lou/C and Wistar rats. A) Representative Western blot of megalin in choroid plexus. B) Representative Western blots of STAT3 and pSTAT3 in hypothalamus. C) Schematic representation of leptin receptor activation in POMC neurons of the ARC nucleus of the hypothalamus. D) Representative Western blots of FoxO1 in hypothalamus. All Western blots were revealed using horseradish peroxidase-conjugated secondary antibodies and enhanced chemiluminescence detection. The quantifications were performed using the ChemiDoc^TM^ XRS and the Quantity One^TM^ software. Results obtained 30 minutes after a leptin challenge of 2 mg/kg i.p., were normalized with actin protein expression. Values are mean + SEM of 4 to 5 animals per group. *p<0.05 compared to Wistar rats using the Student’s *t* test.

With regard to leptin transport through the BBB, it has been shown to be impaired by increased plasma triglyceride (TG) levels, leading to leptin resistance [14,15]. The decreased plasma TG levels of Lou/C rats observed in the present (Table 2), as well as in our previous studies [4,5] could therefore play a role in improving leptin sensitivity through an enhancement of leptin transport across the BBB. Another approach to evaluate the leptin transport process consists in measuring the protein expression of leptin transporters in the choroid plexus. The short leptin receptor isoform (Ob-Ra) was reported to function as a transporter. However, previous results using *in vitro* autoradiography have reported similar gene expression and protein content of Ob-R (probably Ob-Ra and its long receptor isoform Ob-Rb) in the mid and lateral portion of the choroid plexus of Lou/C and Wistar rats [30]. Megalin is another protein described to be involved in leptin transport through the BBB, as well as in the kidney [31,32]. The protein expression of megalin was therefore determined by Western blots on proteins extracted from the dissected choroid plexus of Wistar and Lou/C rats, 30 min after i.p. leptin injection. As can be seen on Figure 3A, no significant difference was observed between the two strains.

The next step consisted in the evaluation of some parameters involved in hypothalamic leptin binding and signaling through its Ob-Rb receptor isoform. We first observed that the expression of Ob-Rb in the whole hypothalamus was similar in the two groups under basal conditions (100.0 + 21.4% and 104.8 + 15.7% in Wistar and Lou/C rats, respectively). Under such conditions, no differences in the protein expression levels of STAT3, pSTAT3 and the ratio pSTAT3/STAT3 were detected between Wistar and Lou/C rats (ratio values of 0.147 + 0.015 and 0.178 + 0.003 for Wistar and Lou/C, respectively). However, after a leptin challenge, STAT3 protein levels tended to be lower in Lou/C than in Wistar rats, whereas pSTAT3 levels were similar in both groups (Figure 3B). This led to an increased pSTAT3/STAT3 ratio in Lou/C rats, suggesting improved leptin signaling in these animals. To corroborate this assumption, protein levels of FoxO1, a transcription factor inhibited by the active pSTAT3 (Figure 3C), were evaluated by Western blots (Figure 3D). As expected, FoxO1 protein levels were significantly lower in Lou/C than in Wistar rats.

**Figure 4:**
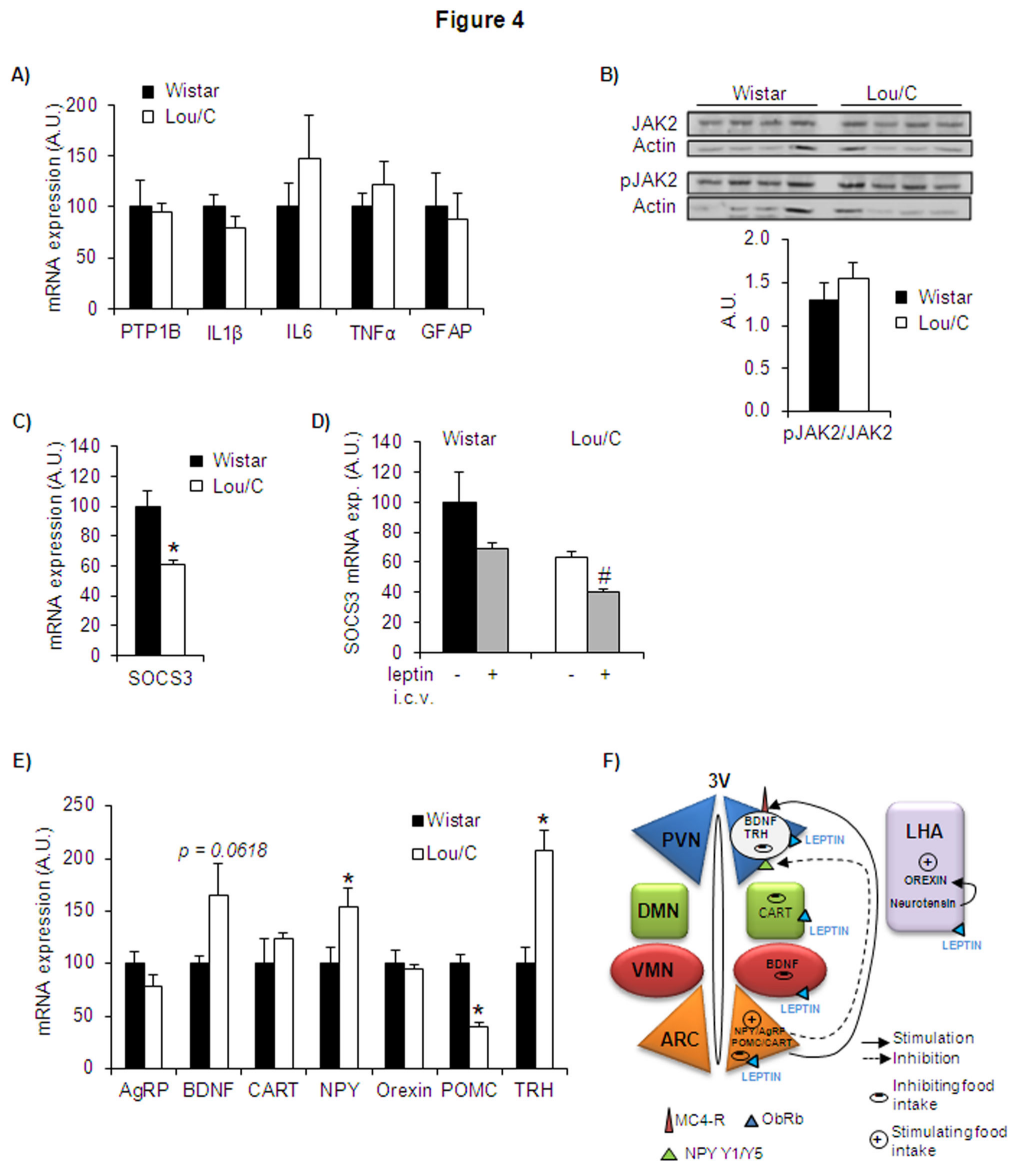
mRNA expression of factors influencing leptin sensitivity and of the main hypothalamic targets of leptin action, in Lou/C and Wistar rats. A) mRNA expression of PTP1B, IL1β, IL6, TNFα and GFAP under basal conditions in the hypothalamus. B) Representative Western blots of JAK2 and pJAK2 revealed using horseradish peroxidase-conjugated secondary antibodies and enhanced chemiluminescence detection. Quantification was performed using the ChemiDoc^TM^ XRS and the Quantity One^TM^ software. Results were normalized with actin protein expression. Values are mean + SEM of 4-5 rats per group. C) mRNA expression of hypothalamic SOCS3 under basal conditions. D) SOCS3 mRNA expression in the hypothalamus of Lou/C and Wistar rats, after an i.c.v. leptin challenge of 10 µg, compared to vehicle injection (NaCl 0.9%). E) mRNA expression of neuropeptides measured in the hypothalamus of Lou/C and Wistar rats under basal conditions and schematic representation of hypothalamic nuclei involved in leptin action. All results were normalized with 36b4 mRNA expression and expressed as percentage of mRNA expression obtained in Wistar rats (100%). Values are mean + SEM of 7-8 rats per group. *p<0.05 compared to Wistar rats using the Student’s *t* test. ^#^p<0.05 compared to vehicle-treated rats using the Student’s *t* test. F) Schematic representation of hypothalamic nuclei implicated. ARC: arcuate nucleus; VMN: ventromedial nucleus; DMN: dorsomedial nucleus; LHA: lateral hypothalamic area; PVN: paraventricular nucleus.

Among the factors modulating leptin sensitivity, the neuronal ER stress was shown to exert a negative influence by markedly inhibiting leptin-induced STAT3 phosphorylation through activation of the protein tyrosine phosphatase 1B (PTP-1B) that inhibits phosphorylation of JAK2 (Figure 3C) [18]. We therefore measured PTP-1B mRNA expression in basal conditions and observed that it was similar in Lou/C and Wistar rats, suggesting that the ER stress is an unlikely candidate underlying the improved leptin sensitivity of the Lou/C group (Figure 4A). This was substantiated by the measurement of JAK2 and activated pJAK2 protein levels, as well as by the pJAK2/JAK2 ratio, which were similar in Lou/C and Wistar rats (Figure 4B).

The same holds true for markers of inflammation, such as IL1β, IL6 and TNFα, as well as for the expression of the glial fibrillary acidic protein (GFAP) taken as an index of gliosis [19] (Figure 4A).

In contrast, we observed that the expression of SOCS3, a transcription factor down-regulating leptin signaling [33], was lower in the hypothalamus of Lou/C compared to Wistar rats in basal conditions (Figure 4C), suggesting a role of SOCS3 in the enhanced leptin sensitivity of the former group. To substantiate this hypothesis, the mRNA expression of SOCS3 was also evaluated 30 min after the acute i.c.v. injection of leptin (10 µg). Whereas they were not modified in Wistar rats (Figure 4D), the leptin-induced SOCS3 mRNA levels were significantly decreased in Lou/C animals, confirming that reduced inhibition of leptin signaling by SOCS3 represents a potential mechanism underlying the increased leptin sensitivity of the Lou/C group.

### Expression of direct and indirect hypothalamic targets of leptin action

Finally, the gene expression levels of the following main hypothalamic targets of leptin were quantified in basal conditions (Figure 4E): POMC, CART (cocaine and amphetamine-regulated transcript), NPY and AgRP (Agouti-related peptide), whose transcription and secretion are directly under the control of leptin in first order neurons of the arcuate nucleus of the hypothalamus [34]; Orexin, whose expression in the lateral hypothalamic area is also dependent on leptin [35]; BDNF, implicated in the anorexigenic leptin effect at the level of the ventromedial hypothalamus [36], and TRH, whose biosynthesis in the paraventricular nucleus of the hypothalamus is directly and indirectly controlled by leptin [22]. In keeping with previous reports [30], Lou/C rats paradoxically exhibited significantly higher NPY and lower POMC mRNA expression levels than Wistar animals, despite lower daily food intake. Interestingly, the mRNA levels of BDNF and of TRH, the factor controlling the hypothalamo-pituitary-thyroid axis involved in the maintenance of energy expenditure, tended or were significantly increased in Lou/C rats (Figure 4E). The mRNA expression of AgRP, CART and orexin was similar in the two strains. Such was also the case for the hypothalamic expression of the most relevant melanocortin receptor, the MC4-R, mediating the effect of AgRP and POMC neurons on second order and downstream neurons (100.0 + 15.3 and 111.0 + 14.3 A.U. in Wistar and Lou/C rats, respectively). A representation of hypothalamic nuclei and leptin signaling pathway is illustrated on Figure 4F.

## Discussion

The Lou/C rat, known as a model of resistance to age- and diet-induced obesity, also exhibits a spontaneous hypophagia. The first aim of the present study was to detail the meal pattern of Lou/C *versus* Wistar rats. Using an equipment allowing for online monitoring of food intake over 24 hrs, we observed that Lou/C rats eat smaller meals, without any decrease in meal numbers, mimicking a situation of higher leptin sensitivity [27]. Sensitivity to the anorexigenic effect of this adipokine was thus investigated in adult animals, compared to age-matched Wistar rats. To this end, different protocols were used, by injecting leptin either centrally or peripherally. In response to i.c.v. leptin injection, food intake was significantly decreased in both Lou/C and Wistar rats at 30 min after the extinction of the lights (1.5 hrs after leptin injection). The effect of leptin was more persistent in Lou/C rats, as the leptin-induced inhibition of food intake lasted for 1hr. In response to peripheral injection, no anorexigenic leptin effect was observed at 30 min after the onset of the dark period, but a maximal effect was seen during the second meal, i.e. 2 hrs after the extinction of the lights, in both Lou/C and Wistar rats. As observed for central injection, leptin-induced hypophagia was more persistent in Lou/C animals and it was also obtained at a leptin dose that was inefficient in the Wistar group (i.e. 1 mg/kg).

Among the parameters that can influence central leptin sensitivity, the first one is leptin transport through the BBB. Indeed and interestingly, a blunted leptin transport across the BBB due to the saturation of Ob-R was suggested in both obese rodents and humans [16,17]. Mitchell and collaborators previously reported no difference between Lou/C and Wistar rats, neither in the expression of Ob-R in the choroid plexus, nor of ^125^I-leptin binding in this brain structure [30]. Megalin, or LRP2 (low-density lipoprotein receptor-related protein 2), is a multi-ligand receptor recently described as being involved in leptin transport across the BBB [31]. In the present study, we observed no difference of megalin protein expression in the choroid plexus of Lou/C *versus* Wistar rats. As proposed by Banks and collaborators, elevated triglyceride levels represent another candidate that can impair leptin transport across the BBB [14]. In our study, triglyceridemia was low in Wistar rats under semi-fasting conditions (around 8 hrs after feeding, at 15: 00 hrs), but it was still lower in the Lou/C group (Table 2). We also previously observed lower triglycerides levels in Lou/C than in Wistar rats in the fed state [4]. These data suggest that one of the factors that can be held responsible for the increased leptin sensitivity in the Lou/C group is their low TG levels. Further investigations are needed to confirm this hypothesis.

After leptin transport within the brain, the next step in leptin action on food intake is its binding to its long receptor isoform, Ob-Rb. In the present study, no difference in Ob-Rb expression in the whole hypothalamus was noted. Data from the literature even reported decreased Ob-Rb expression in the arcuate nucleus (ARC) or the ventromedial nuclei of Lou/C compared to Wistar rats [30]. It is therefore reasonable to conclude that improved leptin sensitivity of Lou/C rats is not linked to Ob-Rb overexpression.

The most interesting result obtained in our study is the increased pSTAT3/STAT3 ratio observed after i.p. leptin treatment in the Lou/C group, as well as the decrease in hypothalamic SOCS3 expression measured under both basal conditions and after a leptin challenge. Similarly, Mitchell and collaborators reported a decreased induction of SOCS3 expression in the ARC of Lou/C rats after i.p. leptin injection [30]. Altogether, these data suggest that the JAK/STAT3 signaling pathway is more efficient in the Lou/C than in the Wistar group, representing one of the factors potentially responsible for their increased leptin sensitivity.

Other candidates for such increased leptin sensitivity of Lou/C rats can also be envisaged. When looking at the hypothalamic neuropeptides which are directly or indirectly modulated by leptin, it was observed that NPY was increased, while POMC was decreased in Lou/C *versus* Wistar rats [8,37]. Such changes cannot therefore be related to the improved leptin sensitivity of Lou/C animals. However, a tendency toward an increase in BDNF, also implicated in the neurotropic role of leptin [38], was observed in Lou/C rats. By checking BDNF receptor expression (p75NTR and TRkB), a significant increase in p75NTR (the low-affinity nerve growth factor receptor) mRNA levels was noted in the hypothalamus of the Lou/C rats compared to the Wistar group (1.8-fold increase, p = 0.0085). The involvement of BDNF in the regulation of metabolism was suggested in the middle of the 90’s. Central infusion of BDNF was reported to induce a severe and dose-dependent decreased appetite in rats [39] and BDNF^+/-^ mice were shown to exhibit chronic hyperphagia and related obesity [40]. More recently, Xu and collaborators suggested that BDNF is a downstream effector, through which the melanocortin system, *via* MC4-R signaling, regulates metabolic homeostasis, particularly in the hypothalamic ventromedial nucleus [41,42]. TRH neurons are also good candidates for the action of BDNF, particularly in the paraventricular nucleus, where TRH neurons highly expressed TRkB. Interestingly, we observed that the mRNA expression of THR, having both thermogenic and anorectic properties, is also higher in Lou/C than in Wistar rats. BDNF and TRH could thus potentially be involved in the improved leptin sensitivity of Lou/C rats, although further investigations, particularly aimed at determining the changes in mRNA expression within specific hypothalamic nuclei, should be performed.

To conclude, spontaneous hypophagia of Lou/C rats appears to be related to improved leptin sensitivity. The main mechanisms underlying such a phenomenon consist in improved leptin signaling through Ob-Rb and consequent overexpression of BDNF and TRH. The low triglyceridemia of Lou/C rats could also be responsible for increased leptin transport through the BBB, although further investigations are needed to confirm this hypothesis. These findings are relevant as they depict one of the mechanisms that can participate to obesity resistance. Furthermore and to our knowledge, the Lou/C rat is the only rat model exhibiting such a resistance, which is encountered in only a few mice models, such as the PTP1B^-/-^ [43], the SOCS3 haploinsufficient mice (SOCS3^+/-^) [44], and the transgenic mice overexpressing Sirtuin 1 in SF1 neurons [45].
